# Genetically predicted chronic rhinosinusitis and the risk of stroke: a two-sample Mendelian randomization study

**DOI:** 10.3389/fneur.2023.1294321

**Published:** 2024-01-11

**Authors:** Jingjing Hu, Hui Wang, Yidan Zhou

**Affiliations:** ^1^Department of Emergency Medicine, Hangzhou Third People’s Hospital, Hangzhou, China; ^2^Department of Geratology, Hangzhou Third People’s Hospital, Hangzhou, China

**Keywords:** stroke, ischemic stroke, intracerebral hemorrhage, Mendelian randomization, rhinosinusitis

## Abstract

**Objective:**

The causal association between chronic rhinosinusitis (CRS) and stroke remains uncertain due to the susceptibility of observational studies to confounding and the possibility of reverse causality. This study aims to examine the potential causal relationship between CRS and the risk of stroke, encompassing various subtypes.

**Methods:**

In this research, we utilized genome-wide association study (GWAS) data for CRS from FinnGen. We identified significant single-nucleotide polymorphisms (SNPs) associated with CRS and used them as instrumental variables (IVs). GWAS data for any ischemic stroke (AIS), ischemic stroke (IS), large-artery atherosclerotic stroke (LAS), small-vessel strokes (SVS), cardioembolic strokes (CES), intracerebral hemorrhage (ICH), lobar ICH, and non-lobar ICH came from multi-ancestry GWAS datasets. We conducted two-sample Mendelian randomization (MR) analyses using inverse variance weighting (IVW), weighted median, and MR-Egger regression methods to investigate potential causal relationships between CRS and stroke. Both heterogeneity and pleiotropy were evaluated by sensitivity analyses.

**Result:**

The IVW analysis revealed no significant associations between CRS and AIS (OR = 0.99, 95% CI [0.93–1.05], *p* = 0.73), IS (OR = 0.97, 95% CI [0.81–1.17], *p* = 0.09), SVS (OR = 0.96, 95% CI [0.82–1.12], *p* = 0.58), LAS (OR = 0.91, 95% CI [0.77–1.08], *p* = 0.09), CES (OR = 0.97, 95% CI [0.81–1.17], *p* = 0.79), ICH (OR = 1.28, 95% CI [0.74–2.22], *p* = 0.28), lobar ICH (OR = 1.22, 95% CI [0.60–2.50], *p* = 0.28), and non-lobar ICH (OR = 1.25, 95% CI [0.65–2.40], *p* = 0.79). Sensitivity analysis found no evidence of horizontal pleiotropy.

**Conclusion:**

According to genetic evidence, this Mendelian randomization (MR) study does not indicate a causal relationship between CRS and stroke in European populations. However, further studies are necessary to comprehensively evaluate the potential association between CRS and stroke.

## Introduction

Chronic rhinosinusitis (CRS) is a common inflammation of the upper airways, posing a significant health concern for 5–12% of the general population (1). Linked to epithelial barrier damage and tissue remodeling, CRS encompasses three distinct inflammatory patterns, according to the EPOS 2020 Guidelines. These include type 1 inflammation targeting viruses, type 2 inflammation targeting parasites, and type 3 inflammation focusing on extracellular bacteria and fungi (2). Ordinarily, the clearance of pathogens restores mucosal barrier integrity. However, in CRS, the compromised mucosa allows the persistent infiltration of external agents, triggering a refractory and unresolved inflammatory response (1). CRS has the potential to give rise to severe intracranial complications, including subdural empyema, cavernous sinus thrombophlebitis, meningitis, and brain abscess (3).

Epidemiological research has revealed robust connections between the incidence of CRS and other inflammatory ailments affecting both the upper and lower respiratory tracts (4–6). Furthermore, a notable link exists between chronic inflammatory diseases and stroke (7, 8). The mechanism between CRS and stroke remains complex and not entirely understood. Several potential explanations have been suggested to contribute to this association. First, close anatomical proximity exists between the sinuses and the intracranial cavity, separated only by a thin bony wall (9). The sphenoid sinus’s lateral wall is mere 0.1 mm thick, and the internal carotid artery lies adjacent to it (10). Such proximity allows for perivascular inflammatory reactions, whether infectious or non-infectious, which could contribute to stroke. Second, CRS can induce vascular issues directly via exposure to inflammatory cytokines and alterations in the coagulation pathway. Elevated levels of proinflammatory cytokines found in sinus fluids have adverse effects on endothelial cell function, promoting atherosclerosis and thrombus formation (11). In immune response and subendothelial smooth muscle cell activation, the responsibility lies with several inflammatory cytokines, such as interleukin-1, interleukin-6, interleukin-17, and C-reactive protein, ultimately expediting the atherogenic process. This leads to premature atherosclerosis, resulting in decreased cerebral blood flow and impaired neural tissue perfusion. Cross-activation of the coagulation pathway may also occur through these inflammatory cytokines, as they can trigger the thrombin coagulation system and enhance the expression of fibrinolytic inhibitory proteins, increasing the likelihood of thrombus formation and thromboembolic events (12, 13). Third, common risk factors shared between CRS and stroke, such as allergies, gastroesophageal reflux, and sleep difficulties, may also contribute to their association. Additionally, treatments for CRS, including corticosteroids and decongestants, may increase cardiovascular and cerebrovascular disease risk. Observational research provides evidence of a connection between CRS and the risk of stroke. Certain investigations have indicated that individuals with CRS may be more prone to experiencing strokes (7, 14, 15). Nevertheless, these observational studies could be constrained by limitations in sample size and the presence of potential confounding factors.

Increasingly popular is Mendelian randomization (MR), a method employing instrumental variable (IV) techniques to estimate causal relationships between genetic risk factors and complex human traits (16). Since exposed IVs are randomly assigned at conception and unaffected by disease status, MR studies can investigate causality between exposure and illness outcomes, mitigating the impact of unobserved confounders and reverse causality (17, 18). In this study, we conducted a comprehensive MR analysis to infer the causality of CRS on stroke based on exposure (CRS) and eight outcomes [any ischemic stroke (AIS), ischemic stroke (IS), large-artery atherosclerotic strokes (LAS), small-vessel strokes (SVS), cardioembolic strokes (CES), intracerebral hemorrhage (ICH), lobar ICH, and non-lobar ICH].

## Methods

### Study design

**Figure 1:**
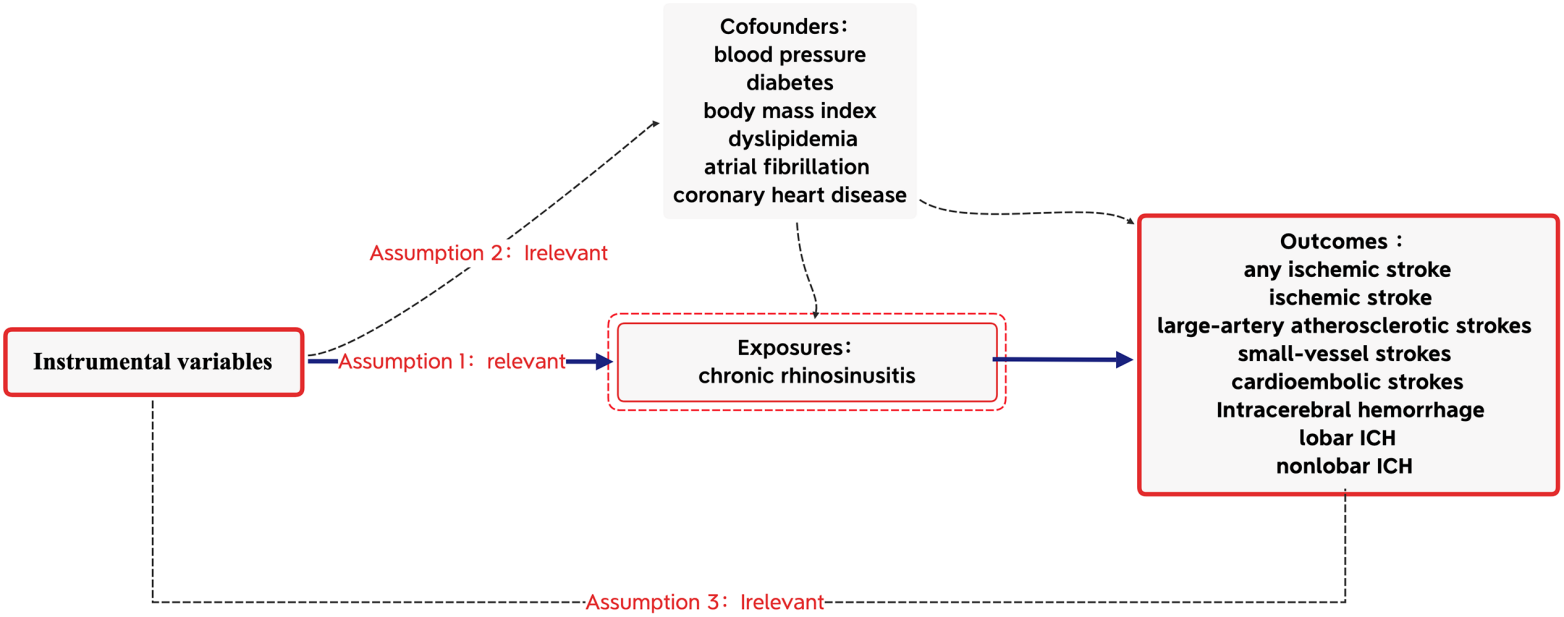
Mendelian randomization model of chronic rhinosinusitis and risk of stroke.

Based on the Strengthening the Reporting of Observational Studies in Epidemiology (STROBE-MR) guidelines, this MR study was conducted (16) to obtain reliable results, as shown in Figure 1. The MR analyses were conducted based on the three core assumptions of instrumental variables (IVs) (11): First that the IVs were linked to the risk factor of CRS; second that the IVs were unrelated to any potential confounders; and third that the IVs were connected to stroke solely through exposures, excluding alternative pathways. All data utilized in this MR analysis were derived from publicly available summary data from the GWAS, with no requirement for additional ethical approval or participant consent as they were previously obtained for each of the original GWAS.

### Data sources

For the exposure dataset, we obtained GWAS data for CRS (finn-b-J10_CHRONSINUSITIS) from FinnGen,^1^ comprising over 260,000 Finnish individuals and nearly 17 million gene variants. FinnGen, initiated in 2017 in Finland, is a public–private research project that integrates imputed genotype data from Finnish biobanks and digital health registries. Detailed information on study design, participants, genotyping, imputation, and quality control methods can be found elsewhere (19).

Stroke data were sourced from a multi-ancestry GWAS, including 67,162 cases and 454,450 controls (20). The majority of individuals were of European descent, comprising 40,585 cases and 406,111 controls. Subtypes of IS included 4,373 LAS, 5,386 SVS, and 7,193 CES. ICH data were sourced from a meta-analysis of six studies that enrolled individuals of European ancestry (21). It included a case cohort of 1,545 individuals (664 lobar and 881 non-lobar cases) and a control cohort of 1,481 individuals. GWAS details are summarized in Table 1.

**Table 1:** Details of the GWASs included in the Mendelian randomization.

Trait	Data source	Population	Sample size	PMID	Phenotypic code
CRS	FinnGen	European	8,524	-	finn-b-J10_CHRONSINUSITIS
Stroke	Malik R et al.	European	40,585	29,531,354	ebi-a-GCST00583
IS	Malik R et al.	European	34,217	29,531,354	ebi-a-GCST005843
LAS	Malik R et al.	European	4,373	29,531,354	ebi-a-GCST005840
SVS	Malik R et al.	European	5,386	29,531,354	ebi-a-GCST005841
CES	Malik R et al.	European	7,193	29,531,354	ebi-a-GCST005842
ICH	Woo D et al.	European	1,545	24,656,865	-
Lobar ICH	Woo D et al.	European	664	24,656,865	-
Non-lobar ICH	Woo D et al.	European	881	24,656,865	-

CRS, chronic rhinosinusitis; IS, ischemic stroke; LAS, large-artery atherosclerotic strokes; SVS, small-vessel strokes; CES, cardioembolic strokes; ICH, intracerebral hemorrhage.

### Genetic instrument selection

Chosen with meticulous care in this study were instrumental variables (IVs), in accordance with rigorous criteria, where SNPs significantly associated with the exposure (*p* < 5e-8) constituted valid IVs. To identify independent instrumental variables (IVs), linkage disequilibrium LD-based SNP clumping was employed, with a clumping r^2^ cutoff of 0.001 and a clumping window of 10,000 kb, utilizing the LD reference panel from the 1,000 Genomes Project. The SNP with the lowest *p*-value was retained (22). The *F* statistics of the instrumental variables were computed to evaluate the degree of weak instrumental bias. The formula used to compute the *F* value was *F* = beta^^^2/se^2, with beta representing the SNP’s effect size and se indicating the standard error of the effect size (23, 24).

### Statistical analysis

We harmonized the exposure and outcome data to ensure that the effect alleles were consistent across exposure and outcome data. The associations between CRS and stroke were ascertained through a two-sample MR analysis employing inverse variance weighted (IVW), weighted median, and MR-Egger regression methods. The primary causal effect estimates were based on the results of IVW; however, it depends on the assumption that all genetic variants serve as valid instrumental variables. The weighted median affords consistent MR estimates if >50% of the weight is from valid SNPs (25). The MR-Egger can detect pleiotropy through its intercept and generate pleiotropy-corrected estimates. However, this method typically has less statistical power (26). To enhance the resulting robustness, we conducted multiple sensitivity analysis studies. The MR-Egger regression has the capability to identify potential pleiotropy and furnish estimates post-correction for pleiotropic effects (26). The MR-PRESSO method is capable of identifying potential outliers and calculating causal estimates after the removal of the identified outliers (27). Cochrane’s Q-derived *p*-value was computed to assess the level of heterogeneity, with *p* < 0.05 indicating the presence of horizontal pleiotropy (28). To detect the directional pleiotropic effect, the value of p for the intercept in MR-Egger was utilized (26). All the analyses mentioned were performed with R software (v4.1.3). We used the TwoSampleMR and MRPRESSO packages (v0.5.6) to perform the MR analysis.

## Results

### Instrumental variable selection

IVs significantly associated with CRS (*p* < 5 × 10–8) were extracted from the GWAS data, and LD (*r*^2^ < 0.001, 10,000-kb) was subsequently removed. Following this, SNPs associated with stroke were obtained from the PhenoScanner database.^2^ We excluded five SNPs (rs1391371, rs1015166, rs3184504, rs4402589, and rs281379) due to their associations with confounding factors (blood pressure, diabetes, body mass index, dyslipidemia, atrial fibrillation, and coronary heart disease). Additionally, palindromic SNPs with a moderate allele frequency were eliminated. The screened SNPs were included in further analyses (Supplementary Data S1–S10). In the IV strength test, no evidence of weak-tool bias was identified (*F*-statistic >10).

### Causal associations between CRS and stroke

**Figure 2:**
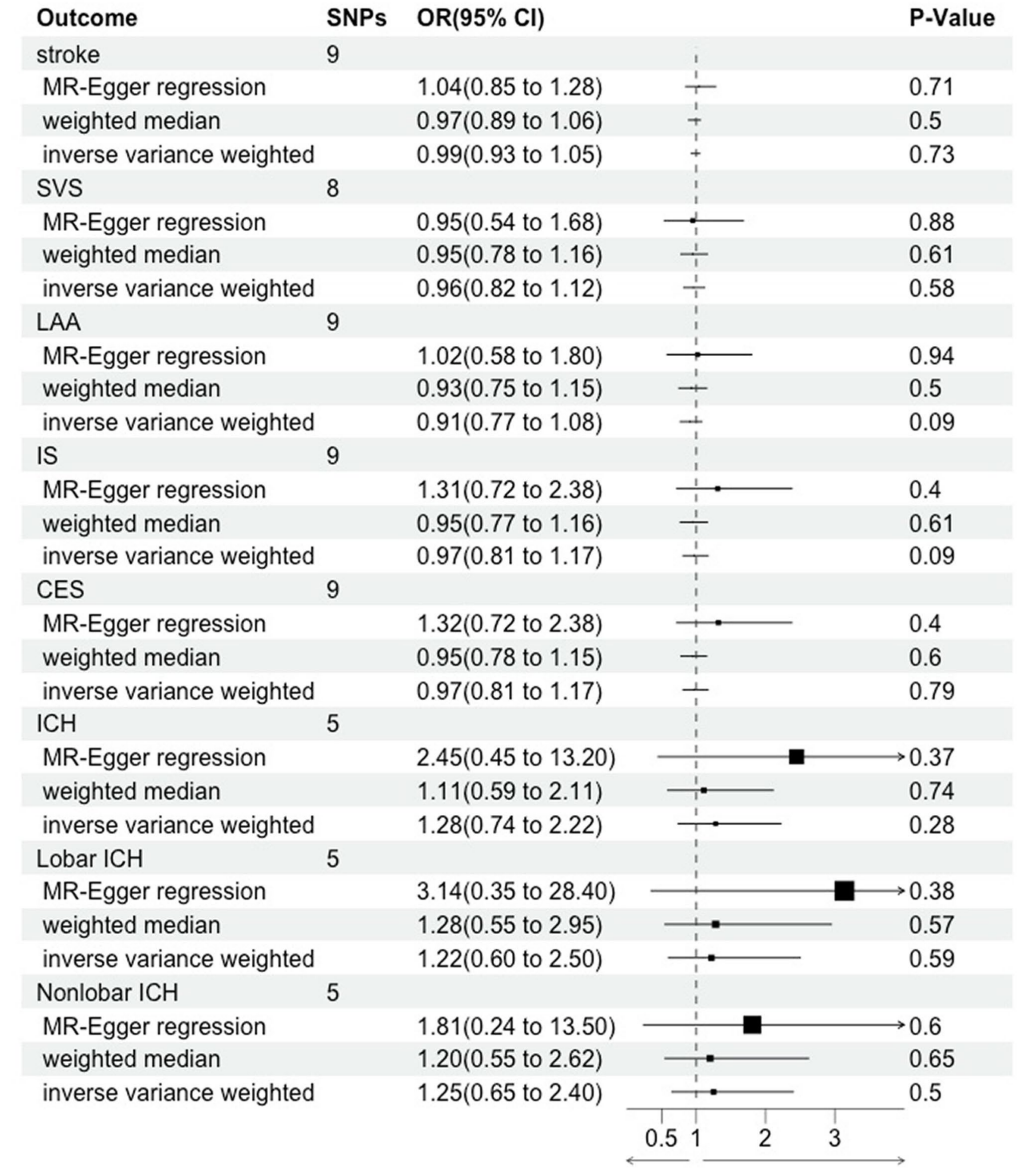
Mendelian randomization assessment of the association between chronic rhinosinusitis and stroke.

The results in the IVW showed no causal correlation between CRS and stroke subtypes (*p* > 0.05). There was no significant difference in the prevalence of stroke (OR = 0.99, 95% CI [0.93–1.05], *p* = 0.73), SVS (OR = 0.96, 95% CI [0.82.1.12], *p* = 0.58), LAA (OR = 0.91, 95% CI [0.77.1.08], *p* = 0.09), IS (OR = 0.97, 95% CI [0.81.1.17], *p* = 0.09), CES (OR = 0.97, 95% CI [0.81.1.17], *p* = 0.79), ICH (OR = 1.28, 95% CI [0.74–2.22], *p* = 0.28), lobar ICH (OR = 1.22, 95% CI [0.60–2.50], *p* = 0.28), and non-lobar ICH (OR = 1.25, 95% CI [0.65–2.40], *p* = 0.79) between CRS patients and controls. Consistent results were obtained through IVW, MR Egger, and weighted median causal association analyses (Table 2; Figure 2).

**Table 2:** Mendelian randomization assessment of the association between chronic rhinosinusitis and stroke.

Outcome	Method	*p*-value	OR (95% CI)	Cochrane’s Q test *p*-value	MR-Egger intercept derived *p*-value	MRPRESSO	SNPs
stroke	MR Egger	0.71	1.04 (0.85–1.28)	0.82	0.62	0.87	9
	Weighted median	0.50	0.97 (0.89–1.06)				
	IVW	0.73	0.99 (0.93–1.05)				
SVS	MR Egger	0.88	0.95 (0.54–1.68)	0.51	0.99	0.54	8
	Weighted median	0.61	0.95 (0.78–1.16)				
	IVW	0.58	0.96 (0.82–1.12)				
LAA	MR Egger	0.94	1.02 (0.58–1.80)	0.95	0.68	0.94	9
	Weighted median	0.50	0.93 (0.75–1.15)				
	IVW	0.09	0.91 (0.77–1.08)				
IS	MR Egger	0.40	1.31 (0.72–2.38)	0.09	0.34	0.17	9
	Weighted median	0.61	0.95 (0.77–1.16)				
	IVW	0.09	0.97 (0.81–1.17)				
CES	MR Egger	0.40	1.32 (0.72–2.38)	0.09	0.34	0.15	9
	Weighted median	0.60	0.95 (0.78)-1.15				
	IVW	0.79	0.97 (0.81–1.17)				
ICH	MR Egger	0.37	2.45 (0.45–13.2)	0.52	0.48	0.61	5
	Weighted median	0.74	1.11 (0.59–2.11)				
	IVW	0.28	1.28 (0.74–2.22)				
Lobar ICH	MR Egger	0.38	3.14 (0.35–28.4)	0.49	0.44	0.52	5
	Weighted median	0.57	1.28 (0.55–2.95)				
	IVW	0.59	1.22 (0.60–2.50)				
Non-lobar ICH	MR Egger	0.60	1.81 (0.24–13.5)	0.69	0.73	0.83	5
	Weighted median	0.65	1.20 (0.55–2.62)				
	IVW	0.50	1.25 (0.65–2.40)				

Mendelian randomized analysis of positive results presented. IS, ischemic stroke; LAS, large-artery atherosclerotic strokes; SVS, small-vessel strokes; CES, cardioembolic strokes; ICH, intracerebral hemorrhage.

### Sensitivity analyses

In the heterogeneity test, all *p*-values from Cochrane’s Q statistics exceeded 0.05, implying the absence of heterogeneity among the SNPs (Table 2). Furthermore, limited evidence of pleiotropy was indicated by the MR-Egger regression intercept in the IVs of CRS with any subtype of stroke. Furthermore, the leave-one-out analysis indicated that a single SNP did not drive the potential causal association between CRS and stroke risk. Forest and funnel plots are shown in Supplementary Figures S1-S5.

## Discussion

This two-sample MR study explored the causal relationship between CRS and IS. This provides evidence that there is no association between stroke and CRS in European populations. To the best of our knowledge, this represents the first Mendelian randomization study on the relationship between CRS and stroke.

Stroke ranks as the second leading cause of disability and mortality worldwide (29). CRS, a recognized chronic disease, is often associated with chronic inflammatory conditions, which are linked to stroke. The mechanism behind this relationship remains complex and not entirely understood. The prevailing hypothesis suggests that the proximity of the paranasal sinuses to the internal carotid artery or the brain could enable the spread of sinus inflammation to the intracranial vasculature. Other hypotheses include cerebral artery spasms, adverse reactions to medications, inflammation-mediated emboli, common risk factors, the side effects of CRS drugs, or complications arising from sinus surgery (30, 31).

Observational studies have indeed supported a link between CRS and IS risk. Lee et al. observed an augmented susceptibility to hemorrhagic stroke (HR = 2.43, 95% CI: 2.10–2.80) and ischemic stroke (HR = 1.76, 95% CI: 1.61–1.92) within the Korean population affected by CRS (7). In a distinct investigation, Jeon et al. discerned a notably heightened prevalence of stroke among individuals with CRS in Korea, as denoted by an adjusted odds ratio of 1.27 (95% CI = 1.15–1.39) (32). Additionally, Kim et al. established a correlation between chronic rhinosinusitis and an elevated incidence of stroke (HR = 1.16, 95% CI: 1.08–1.24) in the Korean population (33). Similar findings were also noted in studies conducted within Taiwan. Kang et al. documented that over a 5-year follow-up period, individuals with CRS exhibited a significantly higher prevalence of ischemic and unspecified stroke (HR = 1.52, 95% CI = 0.94–2.47). Notably, they also found no discernible difference was observed in the prevalence of intracerebral hemorrhage among CRS patients (HR = 0.96, 95% CI = 0.71–1.31) (14). Concurrently, Wu et al. ascertained that patients diagnosed with CRS in Taiwan faced a 1.39-fold increased risk of stroke compared to controls over a 3-year follow-up period (15). However, these studies were retrospective and may have been influenced by urban vs. rural differences, comorbidities, and confounding factors (34). Furthermore, these studies focused on Asian populations, and there may be differences between different populations. Our MR study, which mitigates these issues, may provide more reliable findings. In contrast to their conclusion, our MR study, based on GWAS data from European populations, did not reveal any significant correlation between CRS and stroke. Nevertheless, limitations remain, including the specific population (European) and generalizability to other ethnic groups. Therefore, larger prospective studies and trials based on different ethnicities are required to figure out further issues, such as whether this relationship is causal and the physiopathological of this association. The durations and severities of sinusitis and their impact on stroke should also be taken into consideration. Furthermore, it is necessary to consider whether sinusitis has an impact on the treatment and recovery of stroke.

There are several limitations to this study. First, similar to all MR studies, our findings rely on MR assumptions, which may have inherent limitations. While we performed numerous sensitivity analyses to gage the resilience of our results, we cannot entirely dismiss the potential impact of unmeasured confounding factors on our findings. Second, our study solely utilized a dataset related to CRS, without classified types of CRS. Additional research exploring the impacts of distinct subtypes of CRS would enhance our comprehension of the association between CRS and stroke. Third, our study utilized data from individuals of European ancestry, thus limiting the generalizability of our findings to other ethnic populations. To broaden our conclusion, future research should encompass mixed populations or other ethnic groups.

## Conclusion

Previous retrospective studies based on Asian populations have found a causal relationship between CRS and stroke. Treatments for CRS, including corticosteroids and decongestants, may increase stroke risk. This may have a certain impact on the prescribing tendencies of clinical physicians, especially in the high-risk population for stroke. However, in the MR study, this causal relationship was not observed within the European population. This provides some guidance for clinical medication in CRS. In conclusion, this MR study suggests, at the genetic level, that there is no causal relationship between CRS and stroke in European populations. However, interpreting this result requires caution, and large-scale prospective cohort studies are still needed to confirm it.

## Data availability statement

The original contributions presented in the study are included in the article/Supplementary materials, further inquiries can be directed to the corresponding author.

## Ethics statement

Ethical approval and participant consent were not needed as they were previously obtained for each of the original GWAS. The studies were conducted in accordance with the local legislation and institutional requirements. The participants provided their written informed consent to participate in this study.

## Author contributions

JH: Data curation, Methodology, Writing – original draft. HW: Writing – original draft. YZ: Writing – review & editing.

## Footnotes

^1^https://www.finngen.fi/en

^2^http://www.phenoscanner.medschl.cam.ac.uk/
