# Supplementary figures and images for: Genetically predicted chronic rhinosinusitis and the risk of stroke: a two-sample Mendelian randomization study
Source: Front Neurol. 2024 Jan 11;14:1294321. doi: 10.3389/fneur.2023.1294321 (PMC10808795; doi:10.3389/fneur.2023.1294321)

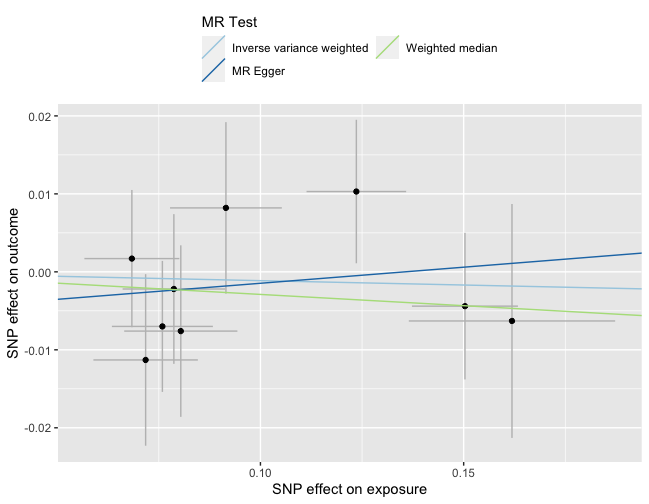


A


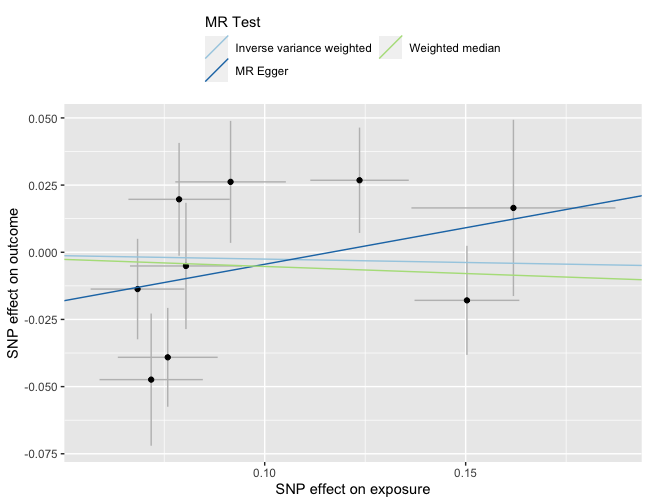


B


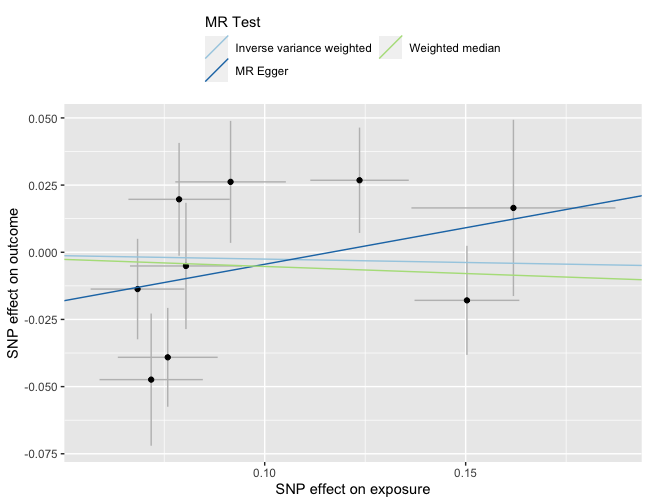


C


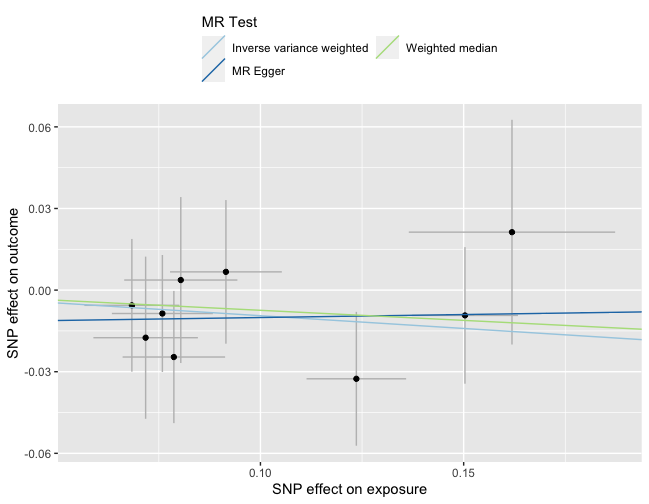


D


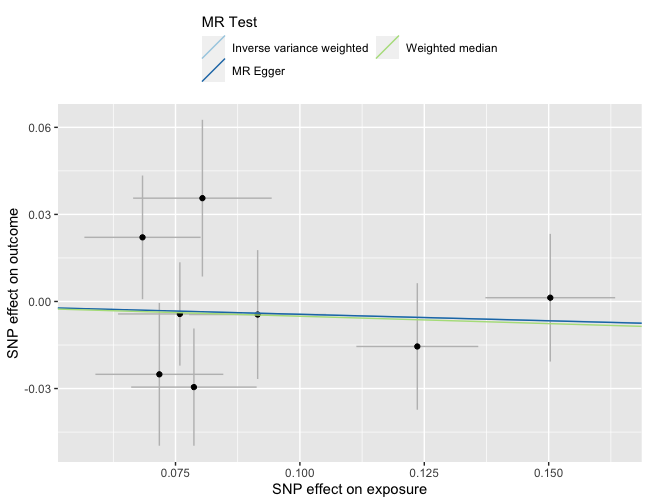


E


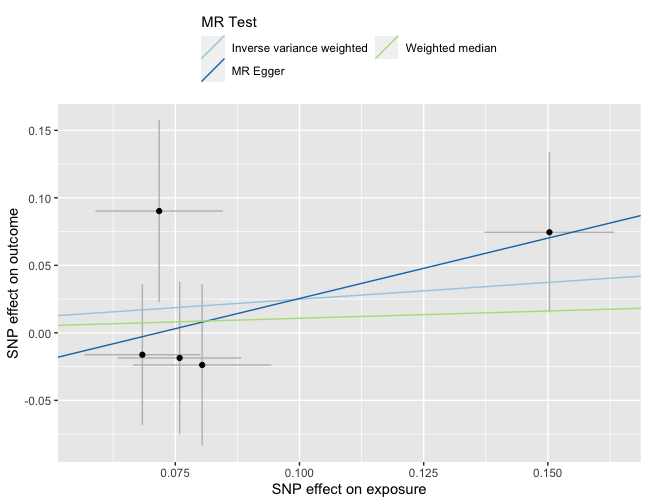


F


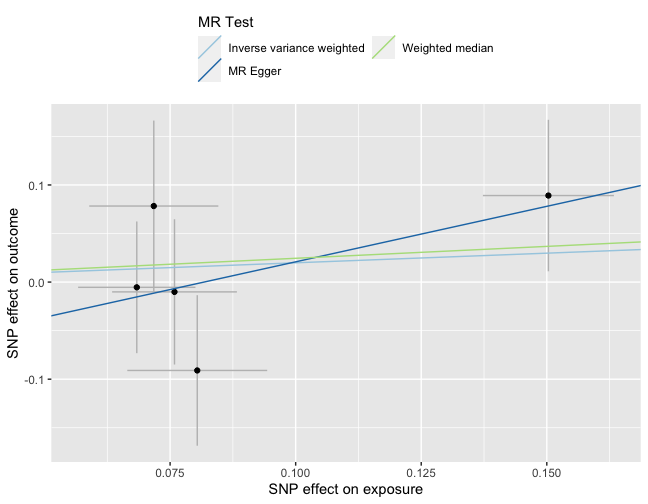


G


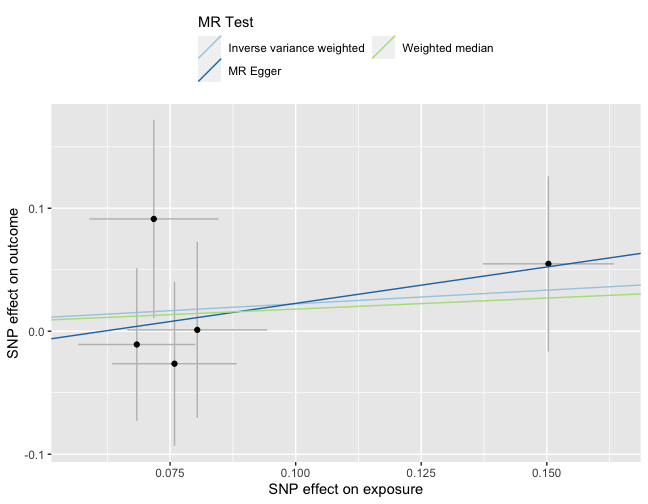


H

Supplement: Supplementary file 2 [file Data_Sheet_2.docx]
